# Correction: Quantifying the contribution of *Plasmodium falciparum* malaria to febrile illness amongst African children

**DOI:** 10.7554/eLife.38361

**Published:** 2018-05-16

**Authors:** Ursula Dalrymple, Ewan Cameron, Samir Bhatt, Daniel J Weiss, Sunetra Gupta, Peter W Gething

Dalrymple U, Cameron E, Bhatt S, Weiss DJ, Gupta S, Gething PW. 2017. Quantifying the contribution of Plasmodium falciparum malaria to febrile illness amongst African children. *eLife* **6**:e29198. doi: 10.7554/eLife.29198.

Published 16, October 2017

The original version of Figure 3 was based on a preliminary set of results that were inaccurate to the final set of results detailed in all other areas of the article. We provide a revised graphic for Figure 3 representing the final results, which is now consistent with the final results presented elsewhere in the article. The final content was presented accurately in all other figures and also in Supplement 1 which provides the numerical dataset used to generate Figure 3. The caption for Figure 3 has also been amended to reflect that the time-series shown is applicable for the years 2006-2014 rather than only the year 2014.

All other content is accurate.

The article has been corrected accordingly.

Corrected Figure 3 is shown here:

**Figure 1:**
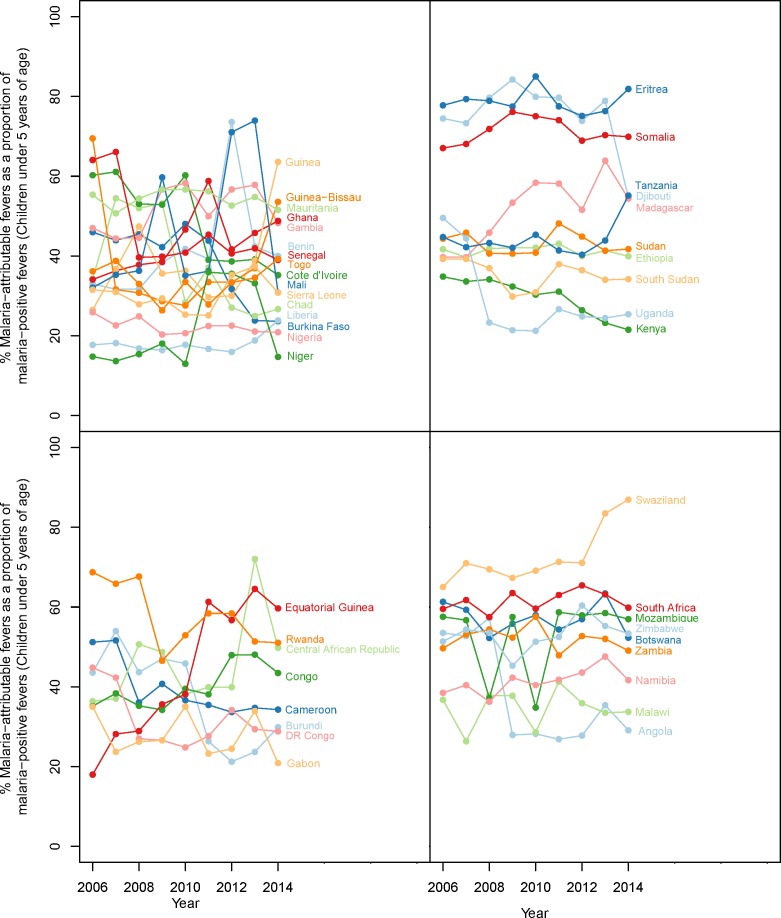


Corrected figure caption: Malaria-attributable fevers as a proportion of malaria-positive fevers (children < 5 years of age).

The originally published Figure 3 is also shown for reference:

**Figure 2:**
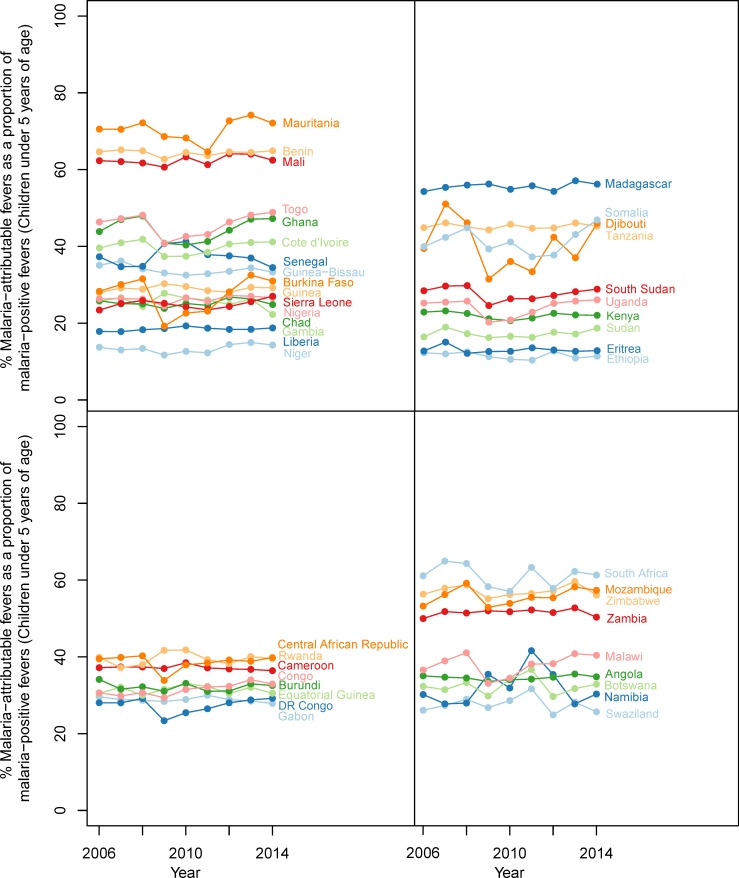


Original figure caption: Malaria-attributable fevers as a proportion of malaria-positive fevers (children < 5 years of age, 2014).

