# Supplementary material for: Quantifying the contribution of Plasmodium falciparum malaria to febrile illness amongst African children
Source: eLife. 2017 Oct 16;6:e29198. doi: 10.7554/eLife.29198 (PMC5665646; doi:10.7554/eLife.29198)
Supplement: Supplementary file 4. [file elife-29198-supp4.docx]

**Supplementary File 4:**

1. Model parameters and credible intervals of the final multinomial model.

| **(Hyper-)Parameter** | **Posterior Mode** | **Approx. SE** |
| --- | --- | --- |
| $\log\kappa_{\mathrm{bg}}$ | 0.02 | 0.06 |
| $\log\tau_{\mathrm{bg}}$ | -1.50 | 0.05 |
| $\log\kappa_{\mathrm{pr}}$ | 1.28 | 0.05 |
| $\log\tau_{\mathrm{pr}}$ | -2.67 | 0.06 |
| $\delta$ | -1.82 | 0.04 |
| $\psi$ | -0.35 | 0.04 |
| $\xi$ | -0.07 | 0.02 |

1. Final model coefficients

|  | **log_kappa_pbg** | **log_tau_pbg** | **log_kappa_pr** | **log_tau_pr** | **logit_prop_maf** | **slope_maf** | **sqslope_maf** |
| --- | --- | --- | --- | --- | --- | --- | --- |
| **Mean** | -1.21954 | -0.16566 | -0.58738 | -0.66011 | -1.51823 | -0.13105 | -0.0047416 |
| **Standard deviation** | 0.114707 | 0.072582893 | 0.131519330 | 0.097987524 | 0.026756679 | 0.021337289 | 0.005256507 |
