# Supplementary material for: Quantifying the contribution of Plasmodium falciparum malaria to febrile illness amongst African children
Source: eLife. 2017 Oct 16;6:e29198. doi: 10.7554/eLife.29198 (PMC5665646; doi:10.7554/eLife.29198)
Supplement: Supplementary file 5. — NMFI is represented here as a fever without a patent malaria infection, MCF as a fever with a patent malaria infection where the fever is caused by a co-infection with an NMFI, and MAF where the malaria infection is the sole cause of the individual's fever. This file is supplementary to Figure 5. [file elife-29198-supp5.pdf]

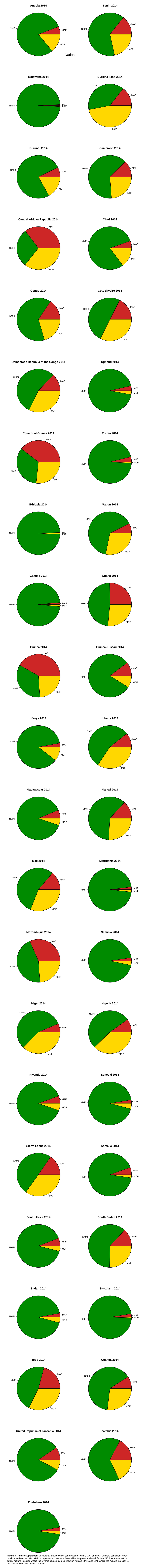

Figure 5 - Figure Supplement 2: National breakdown of contribution of NMFI, MAF and MCF (malaria-coincident fever) to all-cause fever in 2014. NMFI is represented here as a fever without a patent malaria infection, MCF as a fever with a patent malaria infection where the fever is caused by a co-infection with an NMFI, and MAF where the malaria infection is the sole cause of the individual's fever.
